# A Randomized trial of an Asthma Internet Self-management Intervention (RAISIN): study protocol for a randomized controlled trial

**DOI:** 10.1186/1745-6215-15-185

**Published:** 2014-05-24

**Authors:** Deborah Morrison, Sally Wyke, Neil C Thomson, Alex McConnachie, Karolina Agur, Kathryn Saunderson, Rekha Chaudhuri, Frances S Mair

**Affiliations:** 1 General Practice & Primary Care, 1 Horselethill Road, Institute of Health & Wellbeing, University of Glasgow, Glasgow G12 9LX, UK; 2 Deputy Director, Institute of Health and Wellbeing/Interdisciplinary Research Professor, College of Social Sciences, Rm 204, 25-28 Bute Gardens, University of Glasgow, Glasgow G12 8RS, UK; 3 Institute of Infection, Immunity and Inflammation, Gartnavel General Hospital, 1053 Great Western Road, University of Glasgow, Glasgow G12 0YN, UK; 4 Robertson Centre for Biostatistics, Boyd Orr Building, University Avenue, University of Glasgow, Glasgow G12 8QQ, UK

**Keywords:** Asthma, Self-management, Adherence, E-health, Randomized controlled trial, Complex intervention, Inhaled corticosteroids, Behaviour change

## Abstract

**Background:**

The financial costs associated with asthma care continue to increase while care remains suboptimal. Promoting optimal self-management, including the use of asthma action plans, along with regular health professional review has been shown to be an effective strategy and is recommended in asthma guidelines internationally. Despite evidence of benefit, guided self-management remains underused, however the potential for online resources to promote self-management behaviors is gaining increasing recognition. The aim of this paper is to describe the protocol for a pilot evaluation of a website ‘Living well with asthma’ which has been developed with the aim of promoting self-management behaviors shown to improve outcomes.

**Methods/Design:**

The study is a parallel randomized controlled trial, where adults with asthma are randomly assigned to either access to the website for 12 weeks, or usual asthma care for 12 weeks (followed by access to the website if desired). Individuals are included if they are over 16-years-old, have a diagnosis of asthma with an Asthma Control Questionnaire (ACQ) score of greater than, or equal to 1, and have access to the internet. Primary outcomes for this evaluation include recruitment and retention rates, changes at 12 weeks from baseline for both ACQ and Asthma Quality of Life Questionnaire (AQLQ) scores, and quantitative data describing website usage (number of times logged on, length of time logged on, number of times individual pages looked at, and for how long). Secondary outcomes include clinical outcomes (medication use, health services use, lung function) and patient reported outcomes (including adherence, patient activation measures, and health status).

**Discussion:**

Piloting of complex interventions is considered best practice and will maximise the potential of any future large-scale randomized controlled trial to successfully recruit and be able to report on necessary outcomes. Here we will provide results across a range of outcomes which will provide estimates of efficacy to inform the design of a future full-scale randomized controlled trial of the ‘Living well with asthma’ website.

**Trial registration:**

This trial is registered with Current Controlled Trials ISRCTN78556552 on 18/06/13.

## Background

Asthma is common, affecting an estimated 300 million people worldwide [1]. The financial costs associated with asthma care continue to increase [2], while care remains suboptimal - patients continue to overestimate their asthma control, tolerating more symptoms and greater limitations than necessary [3,4]. Promoting self-management, including the use of asthma action plans, along with regular health professional review has been shown to be an effective strategy leading to improved outcomes including improved quality of life, lower rates of healthcare contacts, and fewer days off work and school, and is a recommendation in worldwide asthma guidelines [5-9]. Self-management support aims to improve outcomes in a number of ways: better recognition of deterioration of symptoms, more appropriate responses to exacerbations, and optimizing adherence to medication [10]. Improving adherence to inhaled corticosteroids is crucial to avoid exacerbations, improve day to day control, and reduce the risk of hospitalization and death [11]. Adherence to treatments in many chronic illnesses is low and asthma is no exception [12,13]. Research into non-adherence suggests several rationales, but in common with other chronic conditions recurring themes relate to doubts about the need for the medications in the first place, and concerns about potential side-effects of treatments [12,14].

Despite evidence of benefits, guided self-management remains underused [9-11]. Online interactive tools to support asthma self-management in general have been trialed out with the UK and there is increasing evidence that they may be safe and effective, enabling patients to take a more proactive role, improving asthma quality of life scores and symptoms (and in some cases forced expiratory volume in 1 second (FEV_1_)) [15-18], and potentially demonstrating cost-effectiveness [19]. This suggests that making effective use of available technologies may have the potential to increase uptake of self-management behaviors in those with asthma, without additional cost. How best to achieve this is still not clear [20].

### ‘Living well with asthma’ resource development

We developed an online resource ‘Living well with asthma’ which aims to promote optimal self-management behaviors known to lead to improved outcomes. Exploratory focus group discussions with adults with asthma and primary care nurses clarified the key features deemed most important in a website. These data, along with a preceding literature review [20], informed the initial development of a prototype of the website. The actual content of the pages within the website was developed and refined iteratively with input from adults with asthma, practice nurses, general practitioners, a sociologist, human computer interactions researchers, respiratory physicians, and a health psychologist. Further refinement of the content was undertaken using ‘Think aloud’ studies with adults with asthma. These were undertaken by DM, and participants fed back in real time their views on the contents and usability. Initially this was on paper mock-ups of proposed web pages, and latterly on actual ‘Living well with asthma’ webpages. ‘Think aloud’ studies are a recognized method of gaining information about users’ views in real time as they navigate around a website, providing information about usability and feeding into further development and refinement of the website [21,22]. The ‘Living well with asthma’ website was developed as a standalone resource which should complement face-to-face asthma reviews, but does not require health professional involvement.

A full description of the website development will be available in a forthcoming publication.

### Rationale for the evaluation methods

Guidance for the development of complex interventions recommends pilot and feasibility studies prior to formal evaluations [23,24]. A pilot study is a version of the main study that is run in miniature to test whether the components of the main study can all work together [25], whereas a feasibility study is used to estimate important parameters that are needed to design the main study, such as ease of recruitment, standard deviations of outcome measures, and follow-up rates [25,26]. This study aims to address both issues. Feasibility and piloting are essential to ensure that planned progression to a full-scale randomized controlled trial (RCT) is appropriate in the first place and if it can be undertaken with appropriate power to achieve definitive results. This early evaluation must be broad enough to provide information both about how a full-scale RCT may work in practice but should also have consideration for how the resource may be used beyond the evaluation. Feasibility is investigated through measuring recruitment and retention rates and collecting usability data about the website itself. Measuring clinical outcomes will allow for the collection of important data on the efficacy of the intervention and for the estimation of effect sizes in any future larger RCT.

### Study aims

This study aims firstly to assess the feasibility of conducting a RCT of the clinical effectiveness of an online asthma resource aimed at promoting adherence in adults with poorly controlled asthma, using the ‘Living well with asthma’ resource. Secondly, as a pilot study the aim is to provide estimates of recruitment and retention rates, as well as estimates of the variability of clinical and behavioral outcome measures to inform power calculations for a definitive trial. The study will collect a range of information about the way the intervention was used, including but not limited to: the most and least visited pages, the length of time the intervention was accessed, and how often it was accessed. Finally, this pilot and feasibility study also aims to identify any potential problems to be addressed, and allow for further development of the resource, before a further evaluation.

We hypothesize that this intervention, which has been designed with end user involvement and aims to improve adherence to therapy using multiple strategies (educational information, attitudinal arguments, self-monitoring, and reminders), will result in improved symptom control and quality of life measures in adults with asthma.

## Methods/Design

### Ethical approval and trial registration

Ethical approval for this study was granted by the West of Scotland Research Ethics Committee (ref 13/WOS/004) in March 2013. All participants provided written informed consent. The trial was conducted in accordance with national laws, Good Clinical Practice guidelines and the Declaration of Helsinki 2002. This trial is registered with Current Controlled Trials ISRCTN78556552.

### Recruitment, randomization and blinding

Participants are primarily being recruited from primary care practices within the Greater Glasgow and Clyde health board area and from posters in public places. We aim to recruit 50 participants in total. See Table 1 for full eligibility criteria.

**Table 1:** Inclusion and exclusion criteria

**Inclusion criteria**	**Exclusion criteria**
1. Written informed consent	1. Unstable asthma
2. Age 16 years or older	2. Presence of active lung disease other than asthma
3. Diagnosis of asthma by a health professional and duration of asthma symptoms ≥ 1 year.	3. Mental impairment or language difficulties that make informed consent impossible.
4. Asthma Control Questionnaire score (6 questions version) greater than or equal to 1 (suggesting poorly controlled asthma)	4. Terminal illness
5. Ability to access the internet (excluding via smart phone or tablet).	5. Cognitive impairment.

Consenting participants fulfilling our inclusion and exclusion criteria are being randomized using a third party automated telephone interactive voice response system. Participants will self-complete all questionnaires. Due to the nature of the intervention the blinding of the researcher is not practical, however data will be entered and managed by the Robertson Centre for Biostatistics (RCB), University of Glasgow, and the researcher will take no role in this. The data will be analyzed by a researcher blinded to the allocation of the groups.

### Intervention

**Figure 1:**
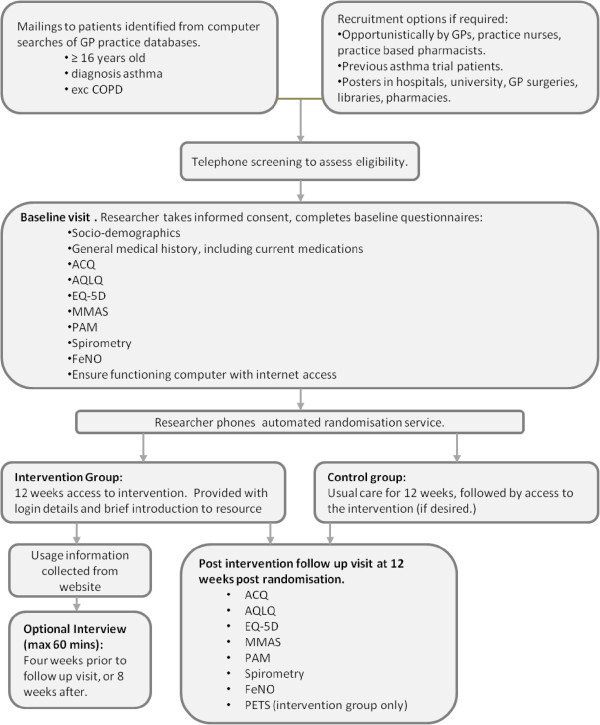
**Study flow chart.** ACQ, Asthma Control Questionnaire; AQLQ, Asthma Quality of Life Questionnaire; FeNO, Fractional Exhaled Nitric Oxide; GP, General Practitioner; MMAS, Morisky Medication Adherence Scale; PAM, Patient Activation Measure; PETS, Problems of Experienced Therapy Scale.

This is a parallel, two arm RCT. See study flow chart (Figure 1) and SPIRIT (Standard Protocol Items: Recommendations for Interventional Trials) checklist (Additional file 1) for further details. The duration of participation is 12 weeks from randomization. Those randomized to the intervention arm will have access to a purpose-built website with the aim of facilitating adherence to asthma medications for 12 weeks. This will include the following five areas: a) allow users to gain understanding of their current degree of asthma control and how they can improve it, specifically by optimizing their use of prescribed medication; b) challenge attitudes and concerns around taking medications for asthma; c) learn how to get the most out of their annual asthma review; d) prompt those who do not have one to seek an asthma action plan to be filled in with a health professional; e) send reminders to participants such as to get the flu vaccine or to order inhalers (participants can opt out of this aspect).

This resource will not advise medication changes directly, but if indicated, may suggest making an appointment with a nurse or doctor for review. Clear advice will be presented for seeking help in an emergency.

### Control group

Those randomized to usual care will be advised to continue to manage their asthma as they would usually. After the follow-up visit at 12 weeks, participants in the control group will be offered 12 weeks of access to the website if they choose.

### Outcomes

#### Primary endpoints

The primary endpoints for this study are: recruitment and retention rates at 12 weeks from baseline, web usability, and changes at 12 weeks from baseline for ACQ [27] and AQLQ [28] scores. Recruitment and retention rates will be measured as well as usage data at 12 weeks, including number of times users logged in and total length of time logged in, number of times individual pages were viewed, and length of time spent on each page.

#### Secondary endpoints

The first secondary endpoint for this study will be changes at 12 weeks from baseline for Patient Activation Measure (PAM) score [29], EQ-5D score (generic measure of health related quality of life) [30], the Morisky Medication Adherence Scale (MMAS) [31], lung function (via pre bronchodilator spirometry FEV_1_, FEV_1_/FVC (forced vital capacity), peak expiratory flow (PEF) performed to American Thoracic Society (ATS) **s**tandards [32], and fractional exhaled nitric oxide levels (FeNO) performed to ATS standards [33].

The second secondary endpoint will be changes in the Problematic Experiences of Therapy Scale (PETS) [34] score at 12 weeks in the intervention group only. The third secondary endpoint will be self-reported healthcare utilization including: routine visit to GP or practice nurse because of asthma, visit to GP because of asthma requiring oral steroids, hospital admission (and length of stay) because of asthma requiring oral steroids, emergency or out of hours’ visit because of asthma requiring oral steroids, and emergency or out of hours’ visit of patients to the GP or GP visit to patient at home because of asthma requiring oral steroids. The fourth secondary endpoint will be self-reported medication utilization including: changes in the level of adherence to prescribed preventer medications, changes to the average number of reliever puffs taken per week, intensification of treatment, or step-up of asthma medications (as defined by British Thoracic Society/Scottish Intercollegiate Guidelines Network (BTS/SIGN) guidelines, and step-down of asthma medications (as defined by BTS/SIGN guidelines).

### Statistical considerations and data handling

While this study is not powered to detect differences in clinical measures, we will report estimates of effect sizes with a 95% confidence interval. The primary objectives include determining recruitment and retention rates to inform the feasibility of running a full-scale trial. We shall recruit 50 participants to estimate these quantities with reasonable precision. For example, if we invite 100 participants to recruit 50, the recruitment rate would be estimated with a confidence interval of ± 10%. Patient characteristics and outcomes will be summarized at baseline, at follow-up, and as changes over baseline. Study groups will be compared using baseline-adjusted linear regression (analysis of covariance (ANCOVA). The effects of baseline and intermediate data on patient outcomes will be explored using linear regression. The variability of outcome data will be used to estimate the sample size required for a definitive study.

### Data management

The RCB, part of the Glasgow Clinical Trials Unit (a fully registered UKCRN Clinical Trials Unit) are managing the randomization procedures and the trial data. Case report forms (CRF) will be used to collect study data. The CRF has been developed by the researcher and the RCB. The RCB are responsible for collating study data.

### Trial management group and patient safety

The routine management of the trial will be coordinated by the trial management group. This will comprise the chief investigator (DM) and four co-investigators (FM, SW, NT, and AM). This group will monitor the progress of the trial to ensure that the protocol is adhered to. This group will meet bimonthly, with monthly recruitment reports via email. Any changes to the study protocol will be following agreement with the trial management group, and subject to approval from Research and Development at NHS Greater Glasgow and Clyde, and the West of Scotland Research Ethics Committee, where required.

The study will end when the trial management group agrees that either the planned sample size has been achieved or the recruitment is so poor that completion of the trial is not feasible.

Only adverse events that are outcome measures will be recorded. All serious adverse events (SAEs) will be recorded at follow-up. All SAEs will be assessed for causality, expectedness, and severity. This assessment is the responsibility of the chief investigator (CI). The trial team will record all SAEs. The CI will endeavor to obtain sufficient information to determine the causality of the adverse event and must provide an opinion of the causal relationship between each SAE and the study intervention. The accumulated SAEs will be sent to the sponsor and the West of Scotland Research Ethics Committee in an annual safety report. Detailed records of all SAEs will be held in the trial master file.

### Annual safety reports

It shall be the responsibility of the trial management group on behalf of the sponsor to submit, once a year throughout the clinical trial, or on request, a safety report to the West of Scotland Research Ethics Committee.

## Discussion

The increasing burden of chronic disease on healthcare providers is well known, and promoting self-care is a strategy for shifting this burden away from healthcare providers. The Internet may provide a cost-effective medium for doing this. Piloting of complex interventions is considered best practice and will maximize the potential of any future full-scale RCT to successfully recruit and be able to report on necessary outcomes. Here we report on feasibility outcomes such as recruitment, retention, and usability of the intervention being investigated, and undertake piloting of an intervention which will aim to determine clinical efficacy.

## Trial status

Recruitment was initiated in June 2013, with the first patient randomized in September 2013, and is ongoing as of February 2014.

## Abbreviations

ACQ: Asthma Control Questionnaire; AQLQ: Asthma Quality of Life Questionnaire; ATS: American Thoracic Society; BTS/SIGN: British Thoracic Society/Scottish Intercollegiate Network; CI: Chief Investigator; CRF: Case Report Form; CTIMP: Controlled Trial of Investigational Medicinal Product; FeNO: Fractional Exhaled Nitric Oxide; FEV_1_: Forced Expiratory Volume in 1 second; FVC: Forced Vital Capacity; GP: General Practitioner; MMAS: Morisky Medication Adherence Scale; PAM: Patient Activation Measure; PEF: Peak Expiratory Flow; PETS: Problems of Experienced Therapy Scale; RCB: Robertson Centre for Biostatistics; RCT: Randomized Controlled Trial; SAE: Serious Adverse Event.

## Competing interests

The authors declare that they have no competing interests.

## Authors’ contributions

DM developed and designed the trial in collaboration with FM, SW, NT, AM, RC. Recruitment of practices or patients was undertaken by DM, KA, KS and FM. Data collection was undertaken by DM, KA and KS. DM wrote the first draft of the protocol and refined it based on comments and feedback from all other authors. All authors read and approved the final manuscript.

## Supplementary Material

Additional file 1 SPIRIT Checklist as appropriate to a non-CTIMP (Controlled Trial of Investigational Medicinal Product). Click here for file

## References

[B1] Masoli M Fabian D Holt S Beasley R Global Initiative for Asthma (GINA) Program The global burden of asthma: executive summary of the GINA Dissemination Committee report Allergy 2004 59 469 478 10.1111/j.1398-9995.2004.00526.x 15080825

[B2] Bahadori K Doyle-Waters MM Marra C Lynd L Alasaly K Swiston J Fitzgerald JM Economic burden of asthma: a systematic review BMC Pulm Med 2009 9 24 10.1186/1471-2466-9-24 19454036 PMC2698859

[B3] Rabe KF Adachi M Lai CKW Soriano JB Vermeire PA Weiss KB Weiss ST Worldwide severity and control of asthma in children and adults: the global asthma insights and reality surveys J Allergy Clin Immunol 2004 114 40 47 10.1016/j.jaci.2004.04.042 15241342

[B4] Haughney J Barnes G Partridge M Cleland J The living & breathing study: a study of patients’ views of asthma and its treatment Prim Care Respir J 2004 13 28 35 10.1016/j.pcrj.2003.11.007 16701634 PMC6750659

[B5] British Thoracic Society British guideline on the management of Asthma Thorax 2008 SupplIV iv1 iv121

[B6] Powell H Gibson PG Options for self-management education for adults with asthma [Systematic Review] Cochrane Database Syst Rev 2002 CD004107 10.1002/14651858.CD004107 PMC8406716 12535511

[B7] Thoonen BP Schermer TR van den Boom G Molema J Folgering H Akkermans RP Grol R van Weel C van Schayck CP Self-management of asthma in general practice, asthma control and quality of life: a randomised controlled trial Thorax 2003 58 30 36 10.1136/thorax.58.1.30 12511716 PMC1746452

[B8] Gibson PG Powell H Wilson A Abramson MJ Haywood P Bauman A Hensley MJ Walters EH Roberts JJL Self-management education and regular practitioner review for adults with asthma [Systematic Review] Cochrane Database Syst Rev 2002 CD001117 10.1002/14651858.CD001117 12535399

[B9] GINA From the Global Strategy for Asthma Management and Prevention, Global Initiative for Asthma (GINA) 2012 Available from: http://www.ginasthma.org/

[B10] Aarne L Guided self management of asthma − how to do it BMJ 1999 319 759 760 10.1136/bmj.319.7212.759 10488007 PMC1116599

[B11] Sims EJ Price D Haughney J Ryan D Thomas M Current control and future risk in Asthma management Allergy Asthma Immunol Res 2011 3 217 225 10.4168/aair.2011.3.4.217 21966601 PMC3178819

[B12] Horne R Compliance, adherence, and concordance: implications for asthma treatment Chest 2006 130 65S 72S 10.1378/chest.130.1_suppl.65S 16840369

[B13] Heaney LG Horne R Non-adherence in difficult asthma: time to take it seriously Thorax 2012 67 268 270 10.1136/thoraxjnl-2011-200257 21685491

[B14] Pound P Britten N Morgan M Yardley L Pope C Daker-White G Campbell R Resisting medicines: a synthesis of qualitative studies of medicine taking Soc Sci Med 2005 61 133 155 10.1016/j.socscimed.2004.11.063 15847968

[B15] van der Meer V Bakker MJ van den Hout WB Rabe KF Sterk PJ Kievit J Assendelft WJ Sont JK SMASHING (Self-Management in Asthma Supported by Hospitals, ICT, Nurses and General Practitioners) Study Group Internet-Based Self-management plus education compared with usual care in Asthma Ann Intern Med 2009 151 110 120 10.7326/0003-4819-151-2-200907210-00008 19620163

[B16] Chan DS Callahan CW Hatch-Pigott VB Lawless A Proffitt HL Manning NE Schweikert M Malone FJ Internet-based home monitoring and education of children with Asthma is comparable to ideal office-based care: results of a 1-year Asthma in-home monitoring trial Pediatrics 2007 119 569 578 10.1542/peds.2006-1884 17332210

[B17] Rasmussen LM Phanareth K Nolte H Backer V Internet-based monitoring of asthma: a long-term, randomized clinical study of 300 asthmatic subjects J Allergy Clin Immunol 2005 115 1137 1142 10.1016/j.jaci.2005.03.030 15940125

[B18] Hashimoto S Brinke AT Roldaan AC van Veen IH Moller GM Sont JK Weersink EJ van der Zee JS Braunstahl GJ Swinderman AH Sterk PJ Bel EH Internet-based tapering of oral corticosteroids in severe asthma: a pragmatic randomised controlled trial Thorax 2011 66 514 520 10.1136/thx.2010.153411 21474498

[B19] Van DMV Van den Hout WB RABE KF Bakker MJ Sterk PJ Assendelft WJ Kievit J Sont JK Cost-effectiveness of internet-based self-management compared with usual care in asthma PLoS One 2011 6 e27108 10.1371/journal.pone.0027108 22096523 PMC3214043

[B20] Morrison D Wyke S Agur K Cameron EJ Docking RI MacKenzie AM McConnachie A Raghuvir V Thomson NC Mair FS Digital Asthma self-management interventions: a systematic review J Med Internet Res 2014 16 e51 10.2196/jmir.2814 24550161 PMC3958674

[B21] Hinchliffe A Mummery WK Applying usability testing techniques to improve a health promotion website Health Promot J Austr 2008 19 29 35 18481929 10.1071/he08029

[B22] Vizri RA Refining the test phase of usability evaluation: how many subjects is enough? Hum Factors 1992 34 457 468

[B23] Medical Research Council Developing and evaluating complex interventions: new guidance 2008 London: MRC

[B24] Public Health Guidance NICE Behaviour Change at Population, Community and Individual Levels 2007 London: NICE

[B25] Arain M Campbell MJ Cooper CL Lancaster GA What is a pilot or feasibility study? a review of current practice and editorial policy BMC Med Res Methodol 2010 10 67 10.1186/1471-2288-10-67 20637084 PMC2912920

[B26] Lancaster GA Dodd S Williamson PR Design and analysis of pilot studies: recommendations for good practice J Eval Clin Pract 2004 10 307 312 10.1111/j..2002.384.doc.x 15189396

[B27] Juniper EF O’Byrne PM Guyatt GH Ferrie PJ King DR Development and validation of a questionnaire to measure asthma control Eur Respir J 1999 14 902 907 10.1034/j.1399-3003.1999.14d29.x 10573240

[B28] Juniper EF Guyatt GH Cox FM Ferrie PJ King DR Development and validation of the mini Asthma quality of life questionnaire Eur Respir J 1999 14 32 38 10.1034/j.1399-3003.1999.14a08.x 10489826

[B29] Hibbard JH Mahoney ER Stockard J Tusler M Development and testing of a short form of the patient activation measure Health Serv Res 2005 40 1918 1930 10.1111/j.1475-6773.2005.00438.x 16336556 PMC1361231

[B30] Group EQ EuroQol–a new facility for the measurement of health-related quality of life: the EuroQol group Health Pol 1990 16 199 208 10.1016/0168-8510(90)90421-9 10109801

[B31] Morisky DE Ang A Krousel-Wood M Ward HJ Predictive validity of a medication adherence measure in an outpatient setting J Clin Hypertens (Greenwich) 2008 10 348 354 10.1111/j.1751-7176.2008.07572.x 18453793 PMC2562622

[B32] Miller MR Hankinson J Brusasco V Burgos F Casaburi R Coates A Crapo R Enright P van der Grinten CP Gustafsson P Jensen R Johnson DC MacIntyre N McKay R Navajas D Pedersen OF Pellegrino R Viegi G Wanger J Standardisation of spirometry Eur Respir J 2005 26 319 338 10.1183/09031936.05.00034805 16055882

[B33] ATS/ERS ATS/ERS recommendations for standardized procedures for the online and offline measurement of exhaled lower respiratory nitric oxide and nasal nitric oxide, 2005 Am J Respir Crit Care Med 2005 171 912 930 15817806 10.1164/rccm.200406-710ST

[B34] Yardley L Kirby S Evaluation of booklet-based self-management of symptoms in meniere disease: a randomized controlled trial Psychosom Med 2006 68 762 769 10.1097/01.psy.0000232269.17906.92 17012531

