# Supplementary material for: A Randomized trial of an Asthma Internet Self-management Intervention (RAISIN): study protocol for a randomized controlled trial
Source: Trials. 2014 May 24;15:185. doi: 10.1186/1745-6215-15-185 (PMC4055289; doi:10.1186/1745-6215-15-185)
Supplement: Additional file 1 — SPIRIT Checklist as appropriate to a non-CTIMP (Controlled Trial of Investigational Medicinal Product). [file 1745-6215-15-185-S1.pdf]

Additional file 1: SPIRIT Checklist as appropriate to a non-CTIMP (http://www.spirit-statement.org/spirit-statement/)

**Title of Study**	Trial Protocol: A pilot Randomized Controlled Trial of Asthma Internet Self Management Intervention: The RAISIN Study.
**Administrative Information**	
**Trial Registration**	Current Controlled Trials – ISRCTN78556552
**Protocol version**	2.1 (22rd October 2013)
**Funding**	Chief Scientist Office (CSO)<br>Ref CAF/11/08
**Roles & Responsibilities**	**Chief Investigator:** Dr Deborah Morrison (Protocol development, recruitment, data collection, data analysis, report writing). [1]<br>**PHD supervisor/Co-applicants:**<br>Prof Frances S Mair(Trial Management Group, protocol development, data analysis, report writing)[2]<br>Prof Sally Wyke (Trial Management Group, protocol development, data analysis, report writing). [3]<br>Prof Neil C Thomson (Trial Management Group, protocol development, data analysis, report writing). [4]<br>Dr Alex McConnachie (Trial Management Group, protocol development, data analysis, supervision of statistical work, report writing). [5]<br><br>**Research Assistants:**<br>Dr Karolina Agur (Protocol development, recruitment, data collection and analysis, report writing).[6]<br>Dr Kathryn Saunderson (Protocol development, recruitment, data collection, report writing).[6]<br>1 – Clinical Academic Fellow (PHD student), General Practice & Primary Care, Institute of Health & Wellbeing, University of Glasgow<br>2- Professor of Primary Care Research, General Practice & Primary Care, Institute of Health & Wellbeing, University of Glasgow<br>3 - Deputy Director, Institute of Health and Wellbeing / Interdisciplinary Research Professor,<br>College of Social Sciences, University of Glasgow<br>4 - Professor of Respiratory Medicine, Institute of Infection, Immunity and Inflammation, University of Glasgow<br>5 - Assistant Director of Biostatistics, Robertson Centre for Biostatistics, University of Glasgow.<br>6 –Clinical Fellow, General Practice & Primary Care, Institute of Health & Wellbeing, University of Glasgow<br><br>**Sponsor:** NHS Greater Glasgow & Clyde, The Tennent Institute, Western Infirmary, Glasgow G11 6NT

**Introduction**	Asthma is a common condition and contributes to significant morbidity and mortality worldwide. Guided self management has been shown to lead to improvements in patient outcomes such as increases in knowledge, confidence and quality of life, as well as reductions in hospitalisations, emergency room visits, unscheduled visits to the doctor, and days off work or school. One mechanism for this is improved adherence, which is known to be low in individuals with asthma. Online resources to promote adherence may have a role in supporting patients to take their medications optimally.
**Background and RA**	This study aims to assess the feasibility of conducting a randomized controlled trial of the clinical effectiveness of an online asthma resourced aimed at promoting adherence in adults with poorly controlled asthma, using the 'Living Well with Asthma' resource.
**Study Centre**	University of Glasgow, NHS Greater Glasgow & Clyde
**Duration of Study**	18 months
**Primary Objective**	To undertake a pilot randomized controlled trial (RCT) of the clinical effectiveness of an Online Asthma Self Management Resource for adults with poorly controlled asthma, also examining feasibility outcomes.
**Primary Endpoints**	1) Recruitment and retention rates at 12 weeks from baseline<br>2) Web use over 12 weeks (via automatically collected data regarding access to website)<br>1) Changes at 12 weeks from baseline for<br>a. Asthma Control Questionnaire (ACQ) [27]<br>b. Asthma Quality of Life Questionnaire (AQLQ) [28]
**Secondary Endpoint**	2) Changes at 12 weeks from baseline for<br>a. EQ-5D [30]<br>b. Patient Activation Measure (PAM) [29]<br>c. Morisky Medication Adherence Scale (MMAS) [31]<br>d. Lung function (via pre bronchodilator spirometry)<br>e. Airway inflammation (via fractional exhaled nitric oxide) [33,35]<br>3) Problematic Experiences of Therapy Scale (PETS) [34] in those in intervention group at follow up visit only (at 12 weeks).<br>4) Self reported health care utilisation<br>5) Self reported medication utilisation<br>6) Adverse events
**Rationale**	Asthma is a common condition and contributes to significant morbidity and mortality worldwide. Guided self management has been shown to lead to improvements in patient outcomes such as increases in knowledge, confidence and quality of life, as well as reductions in hospitalisations, emergency room visits, unscheduled visits to the doctor, and days off work or school. One mechanism for this is improved adherence to medications, which is known to be low in individuals with asthma. Online resources to promote adherence may have a role in supporting patients to take their medications optimally, which is expected to lead to improved symptoms and quality of life.
**Design**	Pilot Randomized Controlled Trial.
**Sample size**	50

**Recruitment**	Potential participants will be aware of the study in the following ways:<br>• Mailings from GP surgeries in Greater Glasgow & Clyde.<br>• Posters in GP surgeries/hospitals/universities/ public spaces such as libraries, pharmacies.
**Randomization**	Via remote automated voice response telephone system
**Main Inclusion Criteria**	• Written informed consent<br>• Age 16 years or older<br>• Diagnosis of asthma by a health professional and duration of asthma symptoms $\geq$ 1 year<br>• ACQ score ≥ 1<br>• Ability to access the internet
**Main Exclusion Criteria**	• Unstable asthma<br>• Presence of active lung disease other than asthma<br>• Mental impairment/ language difficulties making informed consent impossible.<br>• Terminal illness<br>• Cognitive impairment.
**Interventions**	Access to online asthma self management resource which aims to facilitate adherence, through multiple evidence based strategies.
**Duration of treatment**	12 weeks access to intervention
**Statistical Analysis**	Recruitment will be summarised in relation to method of contact; follow-up rates will be summarised for each study group. Factors associated with recruitment and retention will be investigated. Patient characteristics and outcomes will be summarised at each time point. Study groups will be compared using baseline adjusted linear regression (ANCOVA). Potential mediator and moderator variables will be explored using linear regression. The sample size required for a definitive study will be estimated.
